# Comparative Elicitation of Phenolic Accumulation in *Salvia officinalis* Hairy-Root Cultures Using Salicylic Acid, Silver Nitrate, LED Spectra, and Microbial Preparations

**DOI:** 10.3390/biology15181613

**Published:** 2026-09-13

**Authors:** Rahmanifar Behnaz, Aglaia Popa, Oana-Alina Boiu-Sicuia, Oana Livadariu, Călina Petruța Cornea

**Affiliations:** 1 Faculty of Biotechnology, University of Agronomic Sciences and Veterinary Medicine of Bucharest, 59 Mărăști Blvd, District 1, 011464 Bucharest, Romaniaoana.sicuia@bth.usamv.ro (O.-A.B.-S.); oana.livadariu@biotehnologii.usamv.ro (O.L.); petruta.cornea@bth.usamv.ro (C.P.C.); 2 Research-Development Institute for Plant Protection, 8 Ion Ionescu de la Brad Blvd, District 1, 013813 Bucharest, Romania

**Keywords:** *Salvia officinalis*, hairy roots, *Agrobacterium rhizogenes*, phenolics, elicitation, LED light

## Abstract

*Salvia officinalis* L. is a pharmaceutically important species rich in rosmarinic acid, yet strategies to enhance phenolic accumulation in controlled systems remain limited. This study compared three elicitor categories on polyphenol biosynthesis in *Agrobacterium rhizogenes*-transformed hairy root cultures: chemical (salicylic acid, AgNO_3_), physical-Light-emitting diode (LED light spectra: white, blue, red, green, darkness), and biological (*Bacillus velezensis* BPVs2cell lysate and cell-free supernatant, *Trichoderma asperellum* TdCP biomass lysate). Within the chemical-elicitation series, AgNO_3_ produced the strongest response, increasing total phenolic content by 39.2% after 7 days compared with the untreated control and inducing an approximately three-fold increase in rosmarinic acid, together with elevated levels of caffeic, syringic, and chlorogenic acids. Salicylic acid exhibited an inhibitory effect, decreasing total phenolic content by 50%. Exposure to blue and red LED light resulted in a two-fold increase in total phenolics compared with white light and darkness, while green light uniquely stimulated the exudation of phenolics into the culture medium. Among biological elicitors, *B. velezensis* cell-free supernatant (1.5 mL) triggered a 10-fold increase in rosmarinic acid at day 7, whereas *T. asperellum* produced a transient, non-specific response without targeted rosmarinic acid enhancement. These results demonstrate that elicitor type, dose, and exposure time critically determine phenolic accumulation specificity. AgNO_3_, blue and red LED illumination, and *B. velezensis* cell-free supernatant were identified as promising individual treatments in their respective experimental series; however, combined-treatment effects and possible synergism require confirmation in a factorial experiment.

## Simple Summary

Sage is a medicinal plant that contains natural compounds with potential health and protective benefits, but the amount of these compounds can vary when plants are grown outdoors. This study developed a controlled laboratory method for growing fast-growing sage roots and tested whether different light colours, silver nitrate, salicylic acid, and substances produced by beneficial bacteria and fungi could increase the production of these compounds. Silver nitrate was the most effective treatment, markedly increasing the total amount of plant phenolic compounds and producing about three times more rosmarinic acid after seven days. Blue and red light also increased total phenolic content, while green light encouraged the release of these compounds into the surrounding liquid. A liquid produced by *Bacillus velezensis* increased rosmarinic acid production approximately tenfold. Extracts from treated roots slowed the growth of selected bacteria and fungi, particularly *Bacillus spizizenii* and *Sclerotinia sclerotiorum*, although the effects were relatively weak and varied between organisms. These findings show that controlled sage-root cultures could provide a more consistent source of valuable natural compounds for future research and the development of plant-based products.

## 1. Introduction

*Salvia officinalis* L. is a perennial medicinal plant of the *Lamiaceae* family that has been used traditionally for the management of inflammatory, gastrointestinal, metabolic and neurological conditions. Modern pharmacological studies have associated sage preparations with antioxidant, antimicrobial, anti-inflammatory, antidiabetic and neuroprotective activities, although the chemical composition and biological effects may vary according to plant origin, chemotype, developmental stage and extraction procedure [1]. Essential oils and polar extracts of *S. officinalis* have also shown antibacterial and antioxidant properties, supporting the continued investigation of this species as a source of plant-derived bioactive compounds [2,3].

The biological activity of *S. officinalis* is associated with a chemically diverse profile that includes phenolic acids, flavonoids, diterpenes, triterpenes and volatile terpenoids. Among these compounds, rosmarinic acid is particularly important because it combines high abundance in many *Lamiaceae* species with antioxidant, antibacterial, antiviral and anti-inflammatory properties [4]. Rosmarinic acid is formed through the phenylpropanoid pathway from phenylalanine- and tyrosine-derived precursors, and its accumulation is influenced by plant development, tissue type, nutritional conditions and environmental stress. These characteristics make rosmarinic acid a suitable target for metabolic optimisation in plant in vitro cultures.

The direct extraction of specialised metabolites from field-grown plants is affected by seasonality, environmental variation, slow biomass production and fluctuations in metabolite content. Hairy root cultures provide an alternative production platform because they combine rapid root growth, genetic stability, relatively uniform metabolism and the capacity to synthesise compounds associated with differentiated root tissues [5]. Hairy roots induced by *Agrobacterium rhizogenes* can be maintained under controlled nutritional and environmental conditions, making them suitable for the study of specialised metabolism and for the development of scalable production systems.

Although hairy root cultures can produce specialised metabolites without external stimulation, elicitation is frequently used to redirect cellular metabolism towards selected compounds. Elicitors may be chemical, physical or biological and can activate plant defence signalling, oxidative responses and phenylpropanoid metabolism. Their effects are strongly dependent on elicitor identity, concentration, culture age and exposure duration; therefore, an elicitor that is effective in one species or culture system may have a limited or inhibitory effect in another [6].

Chemical elicitation is one of the most established approaches for enhancing specialised-metabolite production in *Salvia* in vitro cultures. In *Salvia miltiorrhiza* hairy roots, several elicitors, including silver ions, methyl jasmonate, salicylic acid, yeast extract, abscisic acid, and other signalling molecules, have been investigated for their effects on phenolic acids and tanshinones. The response is strongly dependent on elicitor identity, concentration, exposure time, and the metabolic pathway considered. For example, different elicitors can preferentially stimulate phenolic-acid or tanshinone biosynthesis rather than producing a uniform increase in all secondary metabolites [7]. Similar treatment-dependent responses have been reported in other *Salvia* species. Methyl jasmonate increased phenolic accumulation and expression of genes associated with the phenylpropanoid pathway in *Salvia* viridis hairy roots, but the response varied according to the compound analysed and the time after elicitation [8]. Studies involving *S. officinalis* and related species have also shown that silver ions, methyl jasmonate, and salicylic acid can modify phenolic-acid production, although the magnitude and direction of the response depend on the experimental system [9,10].

Light quality represents a distinct form of abiotic or physical elicitation because spectral composition can modify photoreceptor signalling, oxidative status, and the expression of genes involved in phenylpropanoid metabolism. In *S. miltiorrhiza*, blue, red, and combined blue–red LED (Light-emitting diode) treatments have been associated with changes in growth and phenolic-acid production, including stimulation of genes involved in the rosmarinic-acid pathway [11]. Responses to LED spectra are nevertheless species-, tissue-, genotype-, and compound-dependent. For example, LED treatments altered the growth characteristics of *Salvia fruticosa* seedlings, but total phenolic content was not significantly affected under the tested conditions [12]. Evidence from related *Lamiaceae* species further indicates that red and blue wavelengths may increase total phenolics or rosmarinic acid, whereas other spectra can produce neutral or opposite responses. This variability means that light treatments should be optimised for the specific species, organ, culture system, and target metabolite rather than being considered universally stimulatory.

Biological elicitation is based on the ability of microbial cells, cell-wall components, secreted metabolites, and culture filtrates to activate plant defence signalling and redirect secondary metabolism. In *Salvia*, fungal elicitors have received considerable attention. Preparations derived from Trichoderma species have been reported to promote hairy-root growth and stimulate specialised-metabolite biosynthesis in *S. miltiorrhiza*, particularly the production of tanshinones [13]. Bacterial elicitation has also been demonstrated in *Salvia*, although the available studies have mainly focused on *S. miltiorrhiza*. A root–bacteria co-culture system involving *Bacillus* sp. enhanced tanshinone production in *S. miltiorrhiza* hairy roots [14]. In a related study, polysaccharide–protein fractions obtained from the plant-growth-promoting rhizobacterium Bacillus cereus promoted both hairy-root growth and tanshinone production [15]. These findings are important because they show that bacterial elicitation does not necessarily require direct colonisation by living bacteria. Cell-wall fractions, bacterial lysates, and soluble extracellular metabolites may all provide signals capable of inducing plant defence responses and secondary-metabolite biosynthesis. The response is expected to depend on the bacterial strain, the type of preparation, the applied dose, and the duration of exposure. However, the response of *S. officinalis* hairy roots to elicitors derived from *B. velezensis* and *T. asperellum* has not been addressed in the same experimental system as the response to chemical and spectral elicitation. The use of bacterial cell lysate and cell-free supernatant is particularly relevant because these preparations allow the elicitor effect to be evaluated without introducing living microbial populations into the hairy-root culture.

Against this background, the present study uses an *Agrobacterium rhizogenes*-transformed *S. officinalis* hairy-root system to examine three experimentally distinct elicitation approaches. The chemical treatments comprise salicylic acid and silver nitrate; the physical treatments comprise white, blue, red, and green LED light, with darkness as the control; and the biological treatments comprise preparations derived from *B. velezensis* BPVs2 and *T. asperellum* TdCP.

The study therefore addresses two related gaps. First, it examines the response of *S. officinalis* hairy roots to microbial preparations from the selected *Bacillus* and *Trichoderma* strains. Secondly, it provides a comparative overview of how chemical, spectral, and microbial elicitation influence root growth, total phenolic content, individual phenolic acids, and antimicrobial activity within the same plant culture platform. Because the three treatment groups were conducted as separate experimental series, the comparison is exploratory and does not test interactions or synergistic effects between elicitor categories.

The aim of this study was to establish and optimise a hairy-root culture system for *S. officinalis* and to examine, in separate experimental series, the effects of culture medium, auxin supplementation, LED spectra, chemical elicitors, and microbial preparations on root growth, biomass accumulation, phenolic composition, and antimicrobial activity. Particular attention was given to rosmarinic acid and related phenolic acids as target metabolites. By comparing the response patterns produced by salicylic acid, silver nitrate, LED spectra, and preparations derived from *B. velezensis* BPVs2 and *T. asperellum* TdCP, the study aimed to identify treatment-specific effects and define priorities for future factorial optimisation experiments.

## 2. Materials and Methods

### 2.1. Plant Material, Hairy-Root Induction and Molecular Confirmation

Seeds and potted plants of *S. officinalis* obtained from the National Agricultural Research and Development Institute, Fundulea, Romania, were used as plant material. Murashige and Skoog (MS) medium (Merck, Darmstadt, Germany) served as the basal medium for in vitro culture, whilst MS, B5 (Merck, Darmstadt, Germany), and Woody Plant Medium (WPM) media (Merck, Darmstadt, Germany) at full and half strength were used for hairy-root cultivation. Sterile seedling preparation, *Agrobacterium rhizogenes* ATCC 15834 cultivation, explant inoculation and co-cultivation, hairy-root development and maintenance, and molecular confirmation were performed in previous research as described by Rahmanifar et al. (2025) [16]. The transgenic status of the hairy roots was confirmed by PCR detection of the *rolB* and *rolC* genes.

### 2.2. Hairy-Root Culture and Experimental Treatments

Hairy roots longer than 2 cm were excised from the explants and transferred to liquid culture. Cultures were maintained in Erlenmeyer flasks containing 75 mL of liquid medium at 25 °C, under agitation at 120 rpm and in darkness. Subculturing was performed approximately every two weeks using fresh medium.

For **physical elicitation**, hairy-root cultures were exposed to white, red, blue, or green LED light at a distance of approximately 12 cm, with darkness used as the control treatment [17]. The cold-white LEDs had a colour temperature of 5000–8300 K, the deep-red LEDs emitted within the 650–670 nm range, the high-blue LEDs emitted within the 460–490 nm range, and the green LEDs emitted within the 500–540 nm. The LED units had a power rating of 18 W, and a voltage of 220 V. Each treatment consisted of 3 independent culture flasks, and each flask was considered one biological replicate. Light intensity and exposure duration were kept constant, and spectral quality was the principal experimental variable. Root length, branching intensity and fresh biomass were recorded. Branching intensity was calculated as the number of lateral branches divided by the length of the main root. The treatments were maintained for 50 days.

**Chemical elicitation** was performed using salicylic acid at 50 µM and silver nitrate at 2.5 ppm. These concentrations were selected based on previously published elicitation studies in *Salvia* species and related culture systems [18]. No preliminary concentration-response experiment was conducted in the present *S. officinalis* hairy-root line. Accordingly, these treatments were used to compare the responses to two literature-informed single concentrations rather than to determine the optimal concentration. Fresh roots were cultured in liquid medium, and the elicitors were added after the cultures had entered the exponential growth phase. Samples were collected before treatment and after 4 and 7 days of incubation. For the chemical-elicitation experiment, untreated hairy roots sampled before elicitation and at the corresponding sampling times served as controls. Root tissue and culture supernatant were analysed separately.

In experiments conducted with *S. officinalis* hairy roots, the effects of **biological elicitors** on secondary-metabolite biosynthesis and accumulation were evaluated using four experimental treatments:

- *Trichoderma asperellum* strain TdCP biomass lysate;
- *Bacillus velezensis* endophytic strain BPVs2 cell lysate;
- Cell-free supernatant obtained from *B. velezensis* BPVs2 culture;
- An untreated control without elicitor.

For the first two treatments, the elicitors were prepared from autoclaved, grinded microbial biomass. Fungal biomass was obtained from two-week-old, submersed cultures, obtained in Potato-Dextrose Broth (Merck KGaA, Darmstadt, Germany) at 25 °C and 150 rpm orbital shaking. The bacterial biomass was obtained from 3-day-old, submersed cultures, obtained in Luria–Bertani broth (Luria/Miller, Carl Roth GmbH + Co.KG, Karlsruhe, Germany) at 28 °C, under orbital shaking at 150 rpm. The biomass was collected by centrifugation, dried at 75 °C, finely ground, and sterilised by autoclaving. The resulting preparations were added to hairy-root suspensions, which were cultivated for 14 days. For biological elicitation, untreated hairy-root cultures without microbial preparation served as controls.

For the cell-free supernatant treatment, bacterial supernatant was obtained from a 3-day-old *B. velezensis* BPVs2 culture, centrifuged, and filtered through a sterile 0.22 µm membrane. The cell-free supernatant was added to the hairy-root cultures at two volumes, 0.75 mL and 1.5 mL, to provide a limited comparison of the response to two amounts of the crude bacterial preparation. Because the concentration of the active compounds in the supernatant was not quantified, these treatments should not be interpreted as a defined concentration-response experiment. The cultures were incubated for 14 days.

The endophytic strain *B. velezensis* BPVs2 had previously been tested for its ability to biosynthesise various bacterial metabolites, including enzymes and lipopeptides, involved in plant biostimulation [19]. It was selected because of its potential usefulness in plant protection and biostimulation. The strain has currently been submitted for patent protection to the State Office for Inventions and Trademarks under Patent Application No. A100364/10.07.2023 as a plant biostimulant. It has also been deposited in an international microorganism collection at NCAIM, Hungary, under accession number NCAIM (P) B 001510.

### 2.3. Extraction and Phytochemical Analyses

The samples were subjected to ultrasound-assisted extraction with 50% ethanol at a sample-to-solvent ratio of 1:10 (*v*/*w*). The extraction was performed for 15 min at room temperature. The resulting suspensions, including root extracts and culture supernatants, were centrifuged and filtered through 0.22 µm syringe filters before analysis.

**Total phenolic content** was determined using the Folin–Ciocâlteu spectrophotometric method [20]. This assay is based on the reduction of phosphomolybdic and phosphotungstic acids by phenolic compounds, resulting in the formation of blue-coloured molybdenum and tungsten oxides. The absorbance of the reaction products was measured at 765 nm. Gallic acid was used as the calibration standard. Standard solutions were prepared at concentrations ranging from 0 to 500 µg/mL and analysed under the same conditions as the samples. The calibration curve was constructed from the absorbance values measured at 765 nm. Results were expressed as gallic acid equivalents. For root samples, the values were expressed per unit mass of root material, whereas the results for culture supernatants were expressed per millilitre of supernatant.

The **phenolic profile** was determined by high-performance liquid chromatography using a Waters 2695 Alliance system coupled with a Waters 2487 UV–VIS detector (Waters Corporation, Milford, MA, USA) [21]. Separation was performed under reversed-phase conditions using a SunFire column with a particle size of 5 µm and dimensions of 3.9 mm × 150 mm. The column temperature was maintained at 40 °C, whilst the analysed samples were maintained at 20 °C. The mobile phase consisted of 0.5% o-phosphoric acid in water as solvent A and acetonitrile as solvent B. According to the experimental record, the gradient began at an A/B ratio of 90/10, changed to 75/25 at 25 min, changed to 10/90 at 40 min, held at 10/90 for 5 min, and changed to 90/10 at 45 min, where it was maintained for an additional 10 min. Chromatograms were recorded at 280 and 300 nm. Phenolic compounds were identified by comparing their retention times with those of injected analytical standards. The compounds considered included gallic acid, chlorogenic acid, caffeic acid, syringic acid, *p*-coumaric acid, rutin, ferulic acid, rosmarinic acid, ellagic acid and quercetin. Each compound was identified by comparison of its retention time with that of an authentic analytical standard analysed under the same chromatographic conditions. Quantification was performed by integrating the area of each chromatographic peak and applying the corresponding calibration curve prepared for each standard.

### 2.4. Antimicrobial Assays

The antimicrobial activity of extracts from untreated and elicited hairy roots was evaluated against *Bacillus spizizenii* ATCC 6633, *Staphylococcus aureus* ATCC 6538, *Escherichia coli* ATCC 25922, and *Bacillus cereus* ATCC 1178. The fungal test organisms were *Sclerotinia sclerotiorum*, *Fusarium* sp., and *Aspergillus* sp. Agar well diffusion assays were performed using 100 µL of each sample. Bacterial plates were incubated overnight at approximately 36 °C, whereas fungal plates were incubated for seven days at approximately 25 °C. Antimicrobial activity was assessed by measuring the inhibition zones around the wells. Each antimicrobial treatment was tested in 2 independent assay replicates.

For the **antibacterial assay**, the microbial strains were grown overnight in liquid Luria–Bertani (LB) medium under orbital shaking. The bacterial suspension was adjusted to an optical density of 0.3 at 600 nm. This suspension was then incorporated into molten Luria–Bertani agar at a ratio of 1:20 (*v*/*v*). Equidistant within the Petri plates, wells were created in the solidified substrate, and filled with 100 µL of hairy-root extract or culture supernatant. The plates were left for approximately 1 h to allow diffusion of the samples into the agar and were subsequently incubated overnight at 36 ± 0.2 °C. Antibacterial activity was determined by measuring the diameter of the clear inhibition zones surrounding the wells. The intensity of the antibacterial effect was assessed using the Manka scale [22].

The **antifungal activity** was tested using Potato Dextrose Agar (PDA). After the medium had been distributed into sterile Petri dishes and solidified, wells were prepared in the agar and filled with 100 µL of the tested hairy-root extracts or culture supernatants. Mycelial discs obtained from a young culture of fungal strains were placed at a defined distance from the wells, approximately 2 cm, to allow the fungal colony to grow towards the treated areas. The plates were first left for approximately 1 h to facilitate diffusion of the samples and were then incubated at 25 ± 0.2 °C for seven days. Antifungal activity was evaluated by measuring the radial inhibition zones or the clear, non-colonised areas surrounding the wells. The inhibitory effect was recorded using the Manka scale [22].

The inhibition-zone measurements were used to compare the antimicrobial effects of extracts and supernatants obtained from untreated and elicited hairy-root cultures. Bacterial and fungal assays were performed using the same sample volume and diffusion principle, whilst the microbial growth medium, incubation temperature, incubation period, and measurement approach were adapted to the organism tested.

### 2.5. Experimental Design and Statistical Analysis

Statistical analyses were performed using Microsoft Excel 2021 with the Analysis ToolPak for one-way analysis of variance. The experiments were conducted using a completely randomised design with three independent biological replicates per treatment (physical, chemical and biological elicitation). Data are presented as means ± standard deviation. For each dataset and sampling point, differences among treatment means were analysed by one-way analysis of variance (ANOVA). When the ANOVA indicated a significant treatment effect, Tukey’s honestly significant difference test was used for post hoc pairwise comparisons. Statistical significance was set at α = 0.05, corresponding to *p* < 0.05. Because the chemical, LED, and biological treatments were evaluated in separate experimental series, the analyses were not intended to test interactions among the three elicitor categories.

## 3. Results and Discussion

### 3.1. Physical Elicitation

LED treatments affected root growth and altered the phenolic profile of *S. officinalis* hairy roots, with the direction and magnitude of the response depending on the light spectrum. Under the experimental conditions used, red light stimulated biomass accumulation and the longitudinal growth of the hairy roots. Similar effects were observed when the roots were maintained in darkness or under blue light. This suggests that light of different wavelengths influences metabolic pathways involved in cell-growth processes. However, the effects of the different illumination treatments on polyphenolic production did not follow the stimulatory pattern observed for hairy-root growth.

The data presented in Table 1 indicate that LED treatments markedly altered the polyphenol content of *S. officinalis* roots at the descriptive level, although the response was clearly dependent on the light spectrum. In the root biomass, blue light produced the highest total-polyphenol content, approximately twice that recorded under white light, which was used as the control. A similar level was observed under red light. This pattern is consistent with scientific literature, which indicates that light is an important elicitor of secondary metabolites and that its effects on phenolic-compound biosynthesis depend on the plant species, organ, light intensity, and spectral quality of the radiation [11,23].

The results obtained under blue and red light are biologically plausible, as numerous studies have shown that these wavelengths can activate phenylpropanoid pathways and promote the accumulation of phenolic compounds with antioxidant and defence-related functions. Within the *Lamiaceae* family, light and other abiotic factors are considered important regulators of phenolic-acid accumulation, including rosmarinic acid, caffeic acid, and salvianolic acids. These compounds are recognised as relevant markers of secondary metabolism in this taxonomic group [4,24]. In addition, in related experimental systems, light exposure has been associated with increased expression of genes involved in rosmarinic-acid biosynthesis. This supports the interpretation that LED illumination can act as a signal for metabolic elicitation, rather than merely as a growth factor [11].

However, comparisons with other in vitro cultures of *Lamiaceae* species show that this response is not universal. In *Agastache rugosa*, white light increased the contents of rosmarinic acid and tilianin, whereas in *Hyptis marrubioides*, red light promoted biomass accumulation and blue light supported rutin accumulation [25]. This variability confirms observations of other research that plant responses to LED illumination are strongly dependent on both the species and the tissue analysed. Consequently, the results cannot be directly extrapolated from one experimental model to another [23].

In this case, the low and relatively constant polyphenol values detected in the supernatant under dark, white, blue, and red light suggest limited release of phenolic compounds into the external medium, with most compounds apparently remaining within the root tissue. The exception was green light, which caused a marked increase in the polyphenol content of the supernatant. This may indicate a modification of tissue permeability, enhanced exudation, or redistribution of metabolites from the roots into the culture medium. Recent literature on transformed-root cultures of *Lamiaceae* species shows that roots can respond differently to light and that, in certain systems, darkness may even represent the optimal condition for polyphenol accumulation [26]. This suggests that, at the root level, photomodulation of secondary metabolism may involve not only biosynthesis but also metabolite transport, compartmentalisation, and exudation processes.

These observations are broadly consistent with reports that red and blue light can stimulate phenolic accumulation in several *Lamiaceae* and medicinal-plant species. However, species-, tissue-, and protocol-dependent responses prevent direct extrapolation between experimental systems [27], in which red or blue light stimulated total polyphenol levels. Although limited in number, similar results concerning the effects of red and blue light on secondary-metabolite accumulation have also been reported for *Salvia przewalskii* [28]. The accumulation of several polyphenolic compounds, including salvianolic acid A, salvianolic acid B, and rosmarinic acid, as well as terpenoids such as dihydrotanshinones, cryptotanshinone, and tanshinone IIA, was stimulated by both blue and red light.

Plant responses to radiation of different wavelengths also vary according to the species, experimental conditions, light intensity, photoperiod, and other environmental factors that may act synergistically or antagonistically on metabolite biosynthesis. Despite the results reported by different researchers for various plant species, the precise mechanisms underlying the effects of different illumination treatments on secondary-metabolite production remain unclear [11].

Regarding the polyphenolic profile of extracts obtained from hairy roots cultivated under illumination with different wavelengths, differences were observed not only in the types of compounds present but also in their respective concentrations (Table 2).

The phenolic profile obtained for *S. officinalis* roots indicated a relatively limited composition, strongly oriented towards phenolic acids, with rosmarinic acid representing the predominant metabolite in most of the analysed treatments. Under the experimental conditions used, gallic acid, syringic acid, *p*-coumaric acid, ferulic acid, and quercetin were not detected. This suggests that physical elicitation selectively favoured the phenylpropanoid branch responsible for the biosynthesis of compounds that are more characteristic of the *Lamiaceae* family, particularly rosmarinic acid, chlorogenic acid, and caffeic acid. This predominance is consistent with literature, which identifies rosmarinic acid as one of the most important and widely distributed polyphenols in the *Lamiaceae* family, with a central role in stress responses and antioxidant activity in these species [4,24].

In the dark-grown control, the profile was dominated by chlorogenic acid, followed by caffeic acid and rosmarinic acid. Exposure to white light led to a reduction in chlorogenic acid, accompanied by an increase in caffeic acid and, particularly, rosmarinic acid. This suggests a redirection of metabolic flux towards the biosynthesis of phenylpropanoid derivatives. Under blue light, the profile was strongly reduced, with chlorogenic acid and rosmarinic acid not detected and only a small amount of caffeic acid present.

By contrast, red and green light favoured the accumulation of rosmarinic acid, reaching 2.873 µg/g and 3.099 µg/g, respectively. Green light was the only treatment that resulted in the production of ellagic acid. Comparative analysis suggests that sage roots do not respond uniformly to light quality but modify their patterns of metabolite production and accumulation according to the plant organ and light spectrum.

Although blue light resulted in the highest total-polyphenol content, the concentrations of the individual compounds included in the phenolic profile were very low or below the detection limit. This suggests that blue illumination may have promoted the synthesis of other phenolic compounds, conjugated phenolics, or bound phenolic fractions that were not included in the analysed profile.

### 3.2. Chemical Elicitation

Chemical elicitation produced contrasting effects. AgNO_3_ increased total phenolic content, whereas salicylic acid reduced total phenolic content under the tested conditions.

Salicylic acid (SA) is an essential component of plant defence mechanisms under stress and plays an important role in various metabolic processes. Recent studies on the mechanisms of action of salicylic acid, jasmonic acid, and certain metal ions have shown that these compounds modulate plant responses to biotic and abiotic stress by activating cellular signalling pathways. These pathways can induce several effects, including stimulation of Ca^2+^ influx into cells, generation of reactive oxygen species (ROS), and activation of enzymes, ultimately promoting the production of secondary metabolites such as alkaloids, flavonoids, terpenoids, and phenolic compounds [29].

When salicylic acid was used, the total polyphenol content in the treated roots was lower than that in the untreated roots for both incubation periods, namely 4 and 7 days. After 4 days of incubation, the total polyphenol content decreased by approximately 12%. After 7 days of incubation, salicylic acid reduced total phenolic content by approximately 50% relative to the untreated control, and a 39% reduction compared with the level recorded after 4 days of incubation with salicylic acid (Table 3).

At the selected AgNO_3_ concentration of 2.5 ppm, total polyphenol content increased by approximately 33.8% after 4 days and by approximately 39.2% after 7 days compared with the untreated roots. The response therefore confirms a stimulatory effect of the tested AgNO_3_ treatment under the experimental conditions used. Because only one AgNO_3_ concentration was evaluated, the present data cannot establish a dose–response relationship, identify the optimal concentration, or determine whether higher or lower concentrations would produce stronger or weaker responses.

The response to salicylic acid was opposite to the expected stimulatory effect of chemical elicitation. Under the conditions used in this study, salicylic acid at 50 µM reduced total phenolic content by approximately 12% after 4 days and by approximately 50% after 7 days. The chromatographic profile indicates that this was not necessarily a complete suppression of phenolic metabolism, because chlorogenic acid and rutin were maintained or slightly increased, whereas rosmarinic acid was clearly reduced. The response therefore appears to represent a selective remodelling of phenolic metabolism rather than a uniform inhibition of all phenolic pathways.

Salicylic acid can activate defence-related signalling involving reactive oxygen species and nitric oxide, and stimulatory effects on phenolic compounds have been reported in *Salvia miltiorrhiza* cell cultures [30]. However, the direction of the response is dependent on species, tissue type, concentration, exposure duration, and culture conditions. Previous studies in *Salvia* have reported positive effects of salicylic acid, but they have also shown that elicitor concentration affects the expression of phenylalanine ammonia-lyase and the accumulation of phenolic compounds [10].

One possible explanation is that the concentration or exposure period used here exceeded the optimal signalling window for this *S. officinalis* hairy-root line. Prolonged or excessive signalling may generate an oxidative burden, activate feedback regulation, or redirect metabolic resources towards other stress-response processes instead of rosmarinic-acid biosynthesis [9]. The present study cannot distinguish between these possibilities because reactive oxygen species, antioxidant capacity, cell viability, phenylalanine ammonia-lyase activity, and rosmarinic-acid synthase expression were not measured. Therefore, the appropriate conclusion is that salicylic acid at 50 µM was not effective for enhancing total phenolic content or rosmarinic-acid accumulation under the tested conditions, rather than that salicylic acid is generally unsuitable as an elicitor in *Salvia*.

The detection of *p*-coumaric acid and quercetin after 4 days but not after 7 days should not be interpreted as complete absence of these compounds at the later sampling point. Time-dependent and compound-specific responses to elicitation have been reported in hairy-root and other plant culture systems, with metabolite accumulation varying according to elicitor exposure time and concentration [8]. The change observed here may therefore reflect transient biosynthesis, metabolic conversion, degradation, redistribution, or variation in the amount of compound available for extraction. Because intermediate sampling points and pathway-level measurements were not included, the specific cause of this change cannot be established from the present data.

At the level of individual compounds, substantial accumulation of rosmarinic acid, syringic acid, caffeic acid, and chlorogenic acid was observed following AgNO_3_ treatment (Table 4). Compared with the untreated control, the rosmarinic acid content was three times higher, whilst syringic acid showed the strongest increase. Caffeic acid and chlorogenic acid also increased substantially, suggesting robust stimulation of the entire phenylpropanoid network and a clear redirection of precursors towards soluble phenolic metabolites. This is the type of response frequently described for Ag^+^-based elicitors, which may be more effective than other inducers in increasing rosmarinic acid and total polyphenol content [9,31].

Compared with the available literature, this result is highly consistent with previous findings. In studies of *Salvia* spp. and other *Lamiaceae* species, Ag^+^ is often one of the most effective inducers of phenolic compounds because it enhances defence responses and increases the biosynthesis of phenolic acids alongside the activation of enzymes involved in the phenylpropanoid pathway [9].

By contrast, the results obtained using AgNO_3_ as an elicitor were consistent with those reported for numerous plant species, including *Salvia* species. The stimulatory effect of AgNO_3_ on tanshinone production has been reported in *Salvia miltiorrhiza* and *S. castanea* [15,18]. Although the precise mechanism underlying this stimulation has not been fully elucidated, the main effect appears to involve modulation of the expression of key genes associated with biosynthetic pathways [32].

Bakhtiar et al. (2026) demonstrated a synergistic stimulatory effect of combined chemical elicitors, including squalene plus pyruvic acid and methyl jasmonate plus coronatine, on the biosynthesis of metabolites of interest in *Glycyrrhiza glabra* [33].

### 3.3. Biological Elicitation

The production of secondary metabolites of interest in several plant species can also be stimulated through the use of biological elicitors of various origins, including components of microbial cells, such as polysaccharides and proteins, microbial endophytes, and plant-growth-promoting rhizobacteria [29,34]. In *Salvia* species, researchers have reported the effects of endophytes, including *Alternaria* sp., *Chaetomium globosum*, *Cladosporium tenuissimum*, *Paecilomyces* sp., *Mucor fragilis*, *Sarocladium kiliense,* and *Trichoderma atroviride*, on biomass accumulation and the levels of metabolites such as tanshinones and salvianolic acids in *S. miltiorrhiza* [35,36]. These effects have been attributed to biologically active compounds of microbial origin that stimulate or inhibit the expression of specific plant genes involved in different metabolic pathways, including those responsible for secondary-metabolite biosynthesis.

The results reported for *S. miltiorrhiza* hairy roots treated with the fungal endophyte *Cladosporium tenuissimum* showed that the fungus stimulated tanshinone production and accumulation by increasing the expression of genes controlling the biosynthesis of these metabolites [35]. Similarly, the use of homogenised and sterilised *Mucor fragilis* biomass as an elicitor for treating *S. miltiorrhiza* hairy roots resulted in increased levels of certain primary metabolites, including amino acids and fatty acids, as well as secondary metabolites such as diterpenoids and phenolic acids [37].

The use of fungal-derived cellular components as elicitors, including polysaccharides obtained from *Trichoderma atroviride* or *Bacillus cereus*, has also produced positive effects on root growth and on the biosynthesis of tanshinones, dihydrotanshinone I, and cryptotanshinone in *S. miltiorrhiza* hairy roots [15].

The bacterial strain used in the experiments had previously been isolated, identified using molecular techniques, including sequencing, and characterised for its ability to synthesise enzymes and other compounds capable of promoting plant growth [19].

Treatment with *B. velezensis* cell lysate had a relatively moderate effect on total polyphenol content (Table 5), and the values recorded on days 7 and 14 remained below those of the untreated control. However, the use of 1.5 mL of bacterial cell-free supernatant resulted in a substantially higher polyphenol content, approximately 58% higher than the control on day 7.

In the supernatants obtained from elicited hairy-root suspensions, total polyphenol levels were extremely low in all treatments compared with the untreated controls. The chromatographic profiles of the phenolic acids supported this observation, suggesting that the plant cells in suspension retained most secondary metabolites intracellularly, with only negligible release into the culture medium.

The present result extends this research by showing that a cell-free preparation from *B. velezensis* can strongly modify phenolic-acid accumulation in *S. officinalis* hairy roots. Nevertheless, the active compound or compounds responsible for the response cannot be identified from the current data. The bacterial supernatant was not chemically profiled, and bacterial elicitation was not accompanied by measurements of reactive oxygen species, defence-related enzymes, or phenylpropanoid-pathway transcripts. Consequently, the tenfold increase should be interpreted as a treatment-specific metabolic response, not as proof that a particular lipopeptide or other bacterial metabolite directly activated rosmarinic-acid biosynthesis.

A decrease was observed on day 7 in roots treated with the cell lysate and 0.75 mL of bacterial cell-free supernatant (Table 6). This suggests partial activation of the phenylpropanoid metabolism, without effective stimulation of the pathway leading to the maximum accumulation of rosmarinic acid. This type of response is typical of microbial elicitors, which can induce metabolic reorganisation but do not always produce a uniform increase in all phenolic compounds [31].

The response to the *B. velezensis* cell-free supernatant was treatment- and time-dependent. The 1.5 mL treatment produced the strongest accumulation of rosmarinic acid and caffeic acid at day 7, whereas the response was substantially weaker at day 14 (Table 6). Direct comparison with other *Salvia* studies remains limited because the elicitor preparations, doses, culture conditions, and sampling schedules were not identical.

The response to *Trichoderma asperellum* TdCP biomass lysate differed from that of the *Bacillus velezensis* supernatant. The fungal preparation increased the overall phenolic background at the earlier sampling point, but it did not produce a comparable increase in rosmarinic acid, and the effect declined by day 14. This pattern is consistent with a transient activation of general defence or oxidative-stress responses rather than a sustained and selective increase in the rosmarinic-acid pathway. Similar treatment-dependent effects have been described for fungal elicitors in *Salvia* hairy-root cultures, where microbial preparations can stimulate growth or specialised metabolism, but the response depends on the fungal species, elicitor fraction, dose, and exposure time [13,31].

Compared with the LED treatments, selected biological elicitors produced larger relative changes in rosmarinic and caffeic acid under the conditions tested. However, this observation should be interpreted cautiously because the biological and LED experiments used different exposure periods and sampling schedules. The present design therefore does not permit a definitive ranking of biological versus LED elicitation. Treatment with *B. velezensis* produced, on day 7, substantially higher levels of rosmarinic acid and caffeic acid than any of the illumination treatments. *T. asperellum* also maintained an active phenolic profile, although its effect was more moderate. These findings suggest that biotic elicitors activate defence pathways and secondary-metabolite biosynthesis more strongly in hairy-root cultures, in a manner dependent on the species, dose, and exposure duration. The biological treatments produced larger relative changes in selected phenolic compounds under the conditions tested. However, because the biological and LED experiments were conducted as separate experimental series with different exposure periods and sampling schedules, these observations represent exploratory comparisons of response patterns rather than a controlled ranking of elicitor efficacy.

The experiments conducted with *S. officinalis* represent the first reported use of elicitors derived from two microbial species with plant-growth-biostimulating properties [38]. The present findings extend previous bacterial-elicitation studies in *S. miltiorrhiza* by showing that a preparation derived from *B. velezensis* can modify phenolic-acid accumulation in *S. officinalis* hairy roots. However, direct comparison with previous studies is limited by differences in plant species, bacterial preparation, dose, culture conditions, and sampling time.

Following treatment with 1.5 mL of *B. velezensis* BPVs2 cell-free supernatant, rosmarinic acid decreased from 25.635 μg/g after 7 days to 0.468 μg/g after 14 days. This pattern indicates a transient elicitation response and may reflect metabolic adaptation, feedback regulation, degradation, redistribution, or a shift towards other defence-related processes [8]. Because only two sampling times were used and pathway-gene expression, enzyme activity, reactive oxygen species, and compound stability were not measured, the cause of this decline cannot be determined from the present experiment.

The stronger response to the cell-free supernatant than to the autoclaved bacterial lysate suggests that soluble extracellular bacterial compounds may have contributed to the elicitation effect. However, the supernatant was not chemically profiled, so the active component cannot be identified. This interpretation is consistent with previous studies showing that *Bacillus* co-culture and bacterial polysaccharide–protein fractions can stimulate specialised-metabolite production in *Salvia* hairy roots [15].

The fungal strain used in the experiments, *T. asperellum* TdCP, had previously demonstrated plant-growth-promoting and plant-protection properties. To the best of our knowledge, *T. asperellum* was used for the first time in experiments investigating the elicitation of secondary-metabolite biosynthesis. The literature reports similar results for other plant species, including *Panax sikkimensis* and *Salvia miltiorrhiza*, but using different *Trichoderma* species, namely *T. atroviride* and *T. harzianum* [27,29].

Regarding the mechanisms of action of biological elicitors, results reported for culture filtrates and microbial extracts from *Bacillus*, *Pseudomonas*, and *Trichoderma* species suggest that these preparations are rich in chitosans, oligosaccharides, polysaccharides, lipids, and small peptides. These compounds can interact with receptors on the surface of treated plant cells or enter the cells directly, thereby affecting the expression of genes involved in cellular defence and secondary-metabolite biosynthesis, including alkaloids, terpenes, iridoids, and phenylethanoids [27,34].

However, biological elicitors, particularly those of fungal origin, can produce negative effects in treated plant cells at certain concentrations or following prolonged exposure. This highlights the need to identify the active microbial components and clarify their mechanisms of action, particularly when their large-scale use as biological elicitors is being considered [29].

The three elicitation approaches investigated in this study produced distinct metabolic response patterns rather than a uniform increase in all phenolic parameters. Because the LED, chemical, and biological treatments were conducted as separate experimental series with different exposure periods and sampling schedules, the results should be interpreted primarily as within-series comparisons. Direct ranking of the three elicitor categories based on absolute metabolite concentrations would therefore be inappropriate.

Chemical elicitation produced the clearest overall enhancement of phenolic accumulation when AgNO_3_ was used. AgNO_3_ increased total phenolic content and stimulated the accumulation of several individual phenolic acids, including rosmarinic and caffeic acids, whereas salicylic acid produced an inhibitory response under the tested conditions.

In the LED experiment, blue and red light increased total phenolic content relative to the white-light control, while red and green light produced the most pronounced changes in the rosmarinic-acid profile. Green light also markedly increased the concentration of phenolic compounds in the culture supernatant, suggesting an effect on metabolite release or exudation rather than only on intracellular biosynthesis.

Biological elicitation showed a different response pattern. The 1.5 mL cell-free supernatant from *B. velezensis* BPVs2 produced the most pronounced rosmarinic-acid accumulation among the biological treatments, particularly after 7 days, whereas *T. asperellum* TdCP biomass lysate induced a stronger but transient increase in the overall phenolic background without a comparable enhancement in rosmarinic acid.

Taken together, the three elicitor categories appear to influence different aspects of the phenolic response. AgNO_3_ was the most effective treatment within the chemical series for broad phenolic enhancement, blue and red LED light were associated with increased total phenolic content, and *B. velezensis* cell-free supernatant was particularly effective for targeted rosmarinic-acid accumulation. These observations do not establish that one category is universally superior because the experiments were not performed under identical exposure durations, doses, or sampling conditions. A future factorial experiment should include the relevant single treatments, combined treatments, and untreated controls under the same culture conditions, with harmonised sampling times. Such a design would allow the independent, additive, and interactive effects of chemical and spectral elicitation to be statistically separated.

A limitation of the present study is that salicylic acid and AgNO_3_ were each evaluated at only one concentration. Although the selected concentrations were supported by previous studies, the design does not allow the optimal dose, concentration threshold, or possible toxicity range to be determined. The two volumes of *B. velezensis* cell-free supernatant provided only a preliminary comparison of crude preparation amounts and were not equivalent to a defined concentration series. Future experiments should evaluate several concentrations of each chemical elicitor and quantify the active components of the bacterial supernatant in order to establish dose–response relationships and compare elicitors more rigorously.

### 3.4. Evaluation of the Antimicrobial Activity of Sage Root Extracts and Supernatants from Elicited Root Cultures

In recent decades, plant extracts have attracted considerable scientific and applied interest, mainly because of their beneficial effects on human health; their ability to inhibit pathogenic or potentially pathogenic microorganisms in various products, including food products; and their capacity to stimulate beneficial microorganisms [39].

Among plant species, particular interest has been directed towards plants of the genus *Salvia*. The metabolic profiles of extracts obtained from different plant organs, including leaves, stems, roots, and flowers, have revealed the presence of essential oils, alkaloids, carbohydrates, and polyphenols, including coumarins, flavonoids, tannins, and phenolic acids, as well as terpenes, steroids, and other compounds [1,39]. These compounds have been associated with various pharmacological properties, including antiproliferative, anti-inflammatory, antinociceptive, antioxidant, and antibacterial effects [40].

Regarding antimicrobial activity, alcoholic extracts obtained from several *Salvia* species have demonstrated both antibacterial activity [1] and antifungal activity [41]. In *S. officinalis*, *S. triloba*, *S. bulleyana*, *S. tomentosa*, *S. sclarea*, and other sage species, the alcoholic or hydroalcoholic extracts tested exhibited bactericidal or bacteriostatic activity against strains of *Bacillus cereus*, *B. mycoides*, *B. subtilis*, *Enterobacter cloacae*, *Escherichia coli*, *Listeria monocytogenes*, *Proteus* sp., *Pseudomonas* sp., *Staphylococcus aureus*, *Streptococcus mutans*, *Salmonella* sp., and other microorganisms [26,39,42].

The results obtained in the present study using unconcentrated extracts from elicited *S. officinalis* hairy roots showed that certain treatments, including silver nitrate, cell-free bacterial supernatant, and autoclaved *Trichoderma* biomass, induced changes in the polyphenolic composition of the sage extracts. These changes resulted in inhibition of the model strain *Bacillus spizizenii* ATCC 6633 (formerly *B. subtilis* subsp. *spizizenii*) (Table 7). The assays were performed comparatively using extracts from unelicited hairy roots and supernatants from the culture medium in which the roots had been grown.

**Figure 1:**
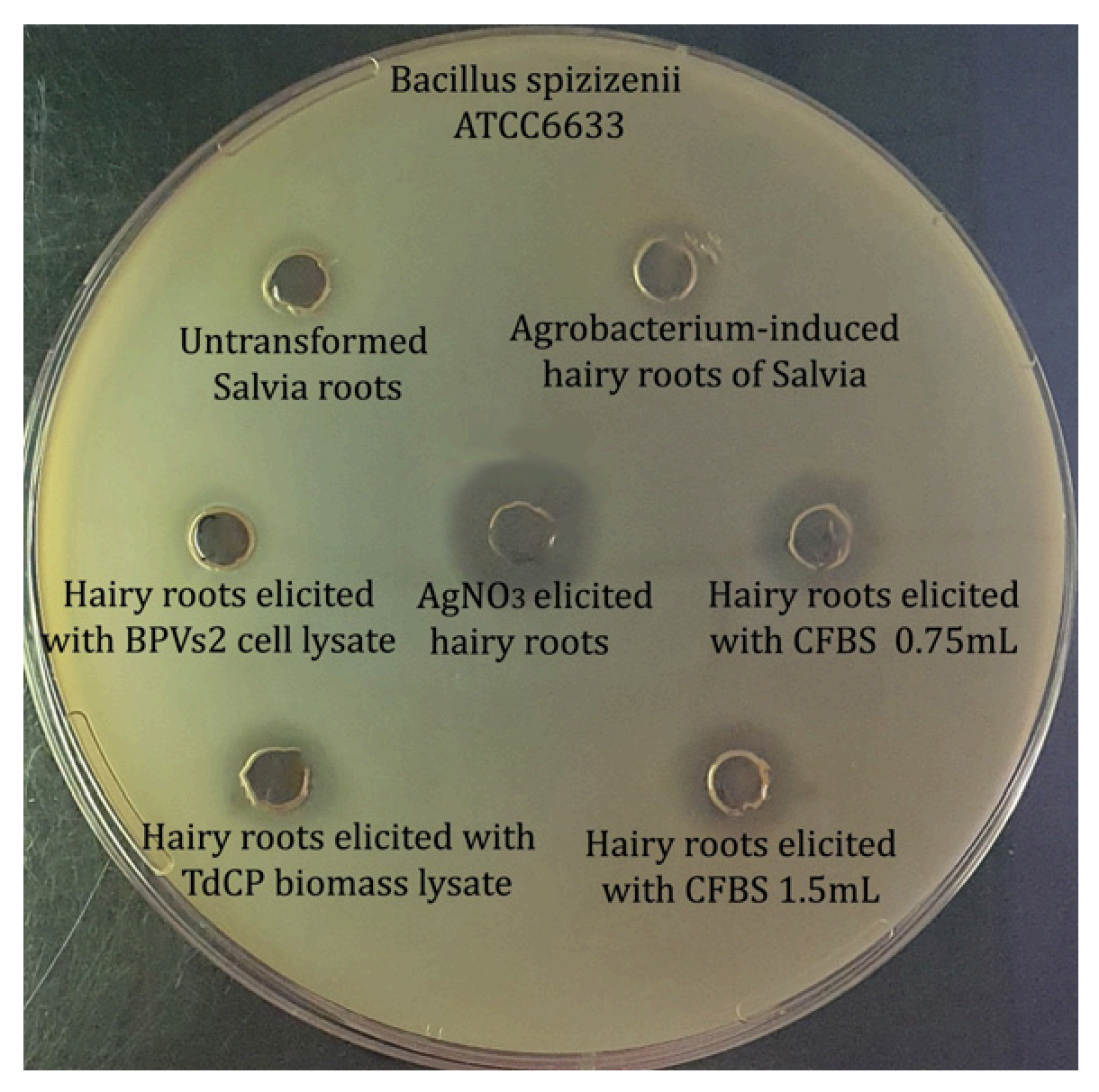
Antibacterial activity of extracts from sage hairy roots elicited with chemical agents (AgNO_3_) or biological agents such as *B. velezensis* BPVs2 cell lysate and cell-free bacterial supernatant (CFBS), or *T. asperellum* TdCP biomass lysate against *B. spizizenii* ATCC 6633.

The antibacterial activity of extracts from elicited hairy roots was clearly evident, particularly following treatment with certain elicitors, whereas no antibacterial activity was detected in the supernatant samples under any of the experimental conditions. Clear zones of growth inhibition against the reference strain *B. spizizenii* ATCC 6633 were observed when bacterial cell-free supernatant was used, with the effect corresponding to the applied dose, as well as following treatment with the other elicitors. The most pronounced results were obtained for extracts from hairy roots elicited with AgNO_3_ (Figure 1). Notably, inhibition zones against this bacterial strain remained visible after several days of incubation. Because viable-cell counts and MBC measurements were not performed, the result is interpreted as sustained growth inhibition rather than confirmed bactericidal activity.

The root-extract samples were also tested against human pathogenic bacteria and quality control strains *Staphylococcus aureus* ATCC 6538, *Escherichia coli* ATCC 25922, and *Bacillus cereus* ATCC 1178.

No clear inhibitory effects were detected against *E. coli* in any of the tested samples. In contrast, a bacteriostatic effect was observed against the two Gram-positive bacterial strains, *S. aureus* ATCC 6538 and *B. cereus* ATCC 1178. After 24 h of incubation, growth-inhibition zones were observed for samples obtained from hairy roots elicited with biological or chemical agents. The largest inhibition-zone diameters were recorded for samples obtained following AgNO_3_ elicitation. Continued incubation for 48 to 72 h resulted in bacterial growth within the inhibition zones. These results indicate time-dependent inhibition of bacterial growth. The observed regrowth within the inhibition zones suggests that a sustained bactericidal effect cannot be concluded from the agar-diffusion assay alone.

**Figure 2:**
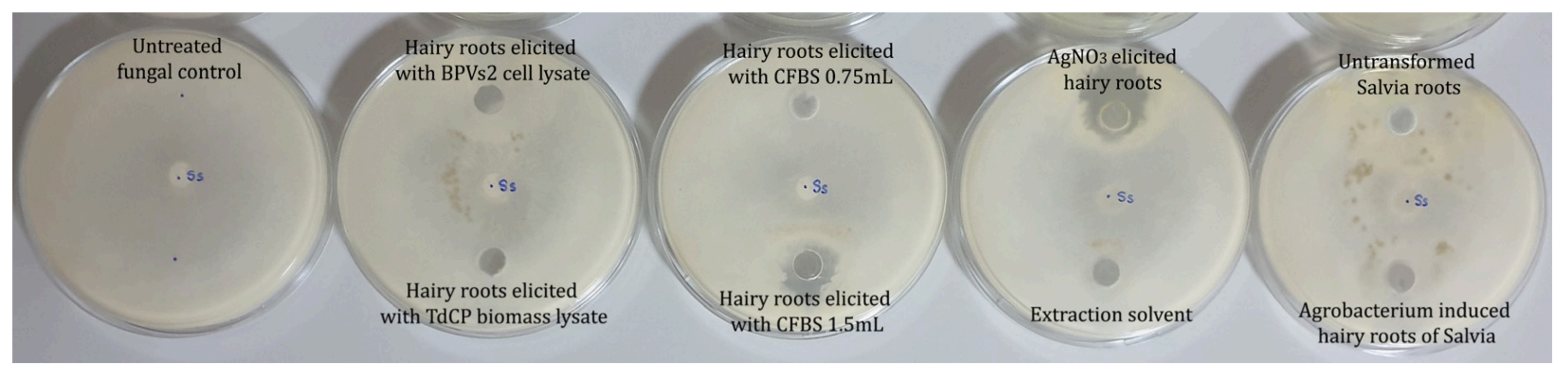
Testing the antifungal activity against *Sclerotinia sclerotiorum* of extracts from *S. officinalis* roots elicited with chemical or biological agents.

The hairy-root extracts were also tested against the filamentous fungi *Sclerotinia sclerotiorum*, *Fusarium* sp., and *Aspergillus* sp. Results revealed a fungicidal effect against *S. sclerotiorum* and a fungistatic effect against *Fusarium* sp. when the hairy-roots had been elicited with cell-free bacterial supernatant or AgNO_3_ (Figure 2).

No inhibitory effects were seen against *Aspergillus* sp., a mycotoxigenic fungus associated with food contamination. The phytopathogenic *Fusarium* sp. tested revealed slight inhibition when exposed to certain hairy-root extracts elicited with AgNO_3_, autoclaved *B. velezensis* biomass and cell-free supernatant. The most sensitive fungal strain tested was *Sclerotinia sclerotiorum* (Table 8).

The agar-well diffusion assay demonstrates inhibition under the tested conditions but does not by itself distinguish bactericidal from bacteriostatic activity or fungicidal from fungistatic activity. The association between phenolic accumulation and antimicrobial activity supports the functional relevance of the elicitation response, but it does not establish that rosmarinic acid or caffeic acid alone caused the observed inhibition. Other extract constituents may also contribute, and the present study did not determine the relative contribution of individual compounds.

The results obtained in the experiments conducted with *S. officinalis* hairy-root extracts were generally consistent with those reported by various researchers for extracts obtained from different *Salvia* species, including elicited and non-elicited hairy-root extracts. In most of the reported studies, concentrated extracts were used, allowing more accurate assessments of inhibitory activity, such as minimum inhibitory concentrations (MICs, expressed in mg/mL).

Regarding the causes of antimicrobial activity, it has been suggested that, in *Salvia* species, this activity is mainly associated with the ability of hairy roots to synthesise and accumulate increased amounts of biologically active secondary metabolites, particularly phenolic compounds, diterpenes, and flavonoids [26]. Hairy-root cultures induced through *Agrobacterium rhizogenes*-mediated genetic transformation represent a stable and efficient biotechnological system for producing bioactive compounds because of their rapid growth, genetic stability, and high level of secondary-metabolite biosynthesis compared with conventional roots [34].

Although many authors consider the antimicrobial activity of sage extracts to be primarily attributable to phenolic compounds, other studies have demonstrated that essential oils produced by *Salvia* species also contribute substantially to these inhibitory effects. For example, Nourozi et al. (2024) [43] reported the antimicrobial activity of biocomponents from *S. sahendica* roots against Gram-positive bacteria, including *Bacillus cereus* and *Staphylococcus aureus*, and weaker activity against Gram-negative bacteria, including *Escherichia coli*, *Salmonella enterica serovar Typhimurium*, and *Pseudomonas* sp. The inhibitory effect was attributed mainly to essential oils present in the extracts.

Similar findings have been reported for other *Salvia* species, including *S. sclarea*, *S. officinalis*, and Mediterranean *Salvia* species, against bacteria, pathogenic yeasts, and filamentous fungi [44]. Inhibitory effects of extracts from different *Salvia* species have been reported against *Candida* species, including *C. albicans*, *C. glabrata*, *C. parapsilosis*, *C. tropicalis*, *C. krusei*, and *C. kefyr* [45,46]. Inhibitory activity has also been reported against filamentous fungi, including *Verticillium dahliae* and *Penicillium aurantiogriseum* [41], *Aspergillus* sp. and *Penicillium* sp. [47], as well as *Botrytis cinerea*, *Fusarium oxysporum*, and *Rhizoctonia solani* treated with leaf extracts of *S. rosmarinus* [48]. According to the available literature, antifungal activity is generally weaker than antibacterial activity.

In addition, Yazici-Tutunis et al. (2025) [49] analysed ethanolic and aqueous extracts from eight *Salvia* species from Anatolia using liquid chromatography high-resolution mass spectrometry (LC-HRMS). They showed that rosmarinic acid was the main component of the extracts, accompanied by substantial amounts of caffeic acid, fumaric acid, salvianolic acid B, and several glucosides. In that study, antimicrobial activity was evaluated in vitro against Gram-positive and Gram-negative bacterial strains, including methicillin-resistant *Staphylococcus aureus* (MRSA), *Enterococcus faecalis*, *Klebsiella pneumoniae*, *Proteus mirabilis*, *Pseudomonas aeruginosa*, and *Bacillus subtilis*. Differences in antibacterial activity were observed depending on the *Salvia* species from which the extracts were obtained. However, for most extracts, the clearest effects were observed against *S. aureus*.

To explain the antibacterial activity, Yazici-Tutunis et al. (2025) [49] associated it with the high content of salvianolic acid, rosmarinic acid, verbascoside, hesperidin, and salicylic acid. Although the molecular mechanisms underlying the antibacterial activity are not yet fully understood, Yazici-Tutunis et al. (2025) [49] evaluated the antibacterial potential of relevant metabolites in silico and demonstrated that these compounds can bind to bacterial target enzymes, including dihydrofolate reductase and dihydropteroate synthase.

Regarding the effectiveness of sage extracts against specific groups of microorganisms, most studies have shown that alcoholic or hydroalcoholic extracts obtained from different vegetative organs of *Salvia* species have stronger inhibitory activity against *Staphylococcus* species, including *S. aureus* and *S. epidermidis*, and weaker activity against Gram-negative bacteria [26,42,45].

The involvement of phenolic compounds accumulated in different plant organs, including roots, leaves, and stems, of *Salvia* species from different geographical regions, including Greece, China, and the Balkans, has been investigated by several researchers [40,44,50]. The association between the accumulation of phenolic compounds, such as caffeic acid, rosmarinic acid, lithospermic acid, and salvianolic acids, and antimicrobial activity has also been reported for several *Salvia* species, including *S. tomentosa*, *S. bulleyana*, and *S. hispanica* [26,42,51].

A considerable proportion of the published results concerning the antimicrobial activity of extracts is based on analyses of root extracts, including hairy-root extracts. In some *Salvia* species, such as *S. bulleyana*, the use of biological elicitors, including yeast extract, or chemical elicitors, such as methyl jasmonate and cadmium chloride, increased the accumulation of phenolic compounds in hairy roots. This had positive effects on antioxidant and cytotoxic activity against tumour cell lines, as well as on antimicrobial activity [26].

Based on a comparison between the published data and the results obtained in the present study, the antimicrobial activity of *S. officinalis* hairy-root extracts against the tested microbial strains can be considered present but relatively weak. However, this activity may be enhanced through the use of eliciting agents, which can stimulate not only root growth but also the accumulation of secondary metabolites. Further studies are therefore required to optimise both the cultivation conditions of the plant material and the extraction and analytical procedures.

## 4. Conclusions

The study established an *Agrobacterium rhizogenes*-mediated hairy-root system for *S. officinalis*. LED treatments influenced phenolic metabolism, with blue and red light increasing total polyphenols, while red and green light favoured rosmarinic acid accumulation. Across the separate experimental series, AgNO_3_ and *B. velezensis* BPVs2 cell-free supernatant produced the strongest responses for selected phenolic outcomes, whereas blue and red LED treatments increased total phenolic content. Because the elicitation categories were not tested in a common factorial design, their relative efficacy and any combined effects remain to be established. In particular, AgNO_3_ at the selected concentration substantially enhanced caffeic and rosmarinic acid levels, while *B. velezensis* BPVs2, particularly at 1.5 mL cell-free supernatant, stimulated pronounced accumulation of these target compounds. However, because only one concentration of each chemical elicitor was tested and the bacterial treatments involved two volumes of an uncharacterised crude supernatant, the optimal doses remain to be determined.

The association between phenolic accumulation and antimicrobial activity supports the functional relevance of the elicitation response, but it does not establish that rosmarinic acid or caffeic acid alone caused the observed inhibition. Other extract constituents may also contribute, and the present study did not determine the relative contribution of individual compounds.

The elicitation-induced changes in phenolic composition were accompanied by improved antimicrobial activity of the hairy-root extracts. AgNO_3_ and 1.5 mL *B. velezensis* BPVs2 cell-free supernatant produced the strongest antibacterial activity against *B. spizizenii* and antifungal activity against *Sclerotinia sclerotiorum*, whereas activity against *Fusarium* and *Aspergillus* was weaker or absent; culture supernatants themselves showed no antibacterial effect. Overall, controlled elicitation enhanced the production of bioactive phenolics and functional activity in *S. officinalis* hairy roots, although further optimisation of cultivation, elicitor dose, exposure time, extraction, and analytical procedures is required.

## Figures and Tables

**Table 1:** Phenolic compound content of roots treated with LED light.

Treatment	Total Polyphenol Content (mg GAE/g Roots or mg GAE/mL Supernatant)
Roots maintained in darkness	0.251 ± 0.012 ^c^
Roots illuminated with white light	0.226 ± 0.011 ^c^
Roots illuminated with blue light	0.451 ± 0.019 ^a^
Roots illuminated with red light	0.438 ± 0.015 ^a^
Roots illuminated with green light	0.351 ± 0.014 ^b^
Supernatant from roots maintained in darkness	0.025 ± 0.012 ^e^
Supernatant from roots illuminated with white light	0.023 ± 0.012 ^e^
Supernatant from roots illuminated with blue light	0.023 ± 0.011 ^e^
Supernatant from roots illuminated with red light	0.025 ± 0.014 ^e^
Supernatant from roots illuminated with green light	0.137 ± 0.013 ^d^

Values are presented as means ± standard deviation from 3 independent biological replicates. Different superscript letters indicate statistically significant differences among treatments according to Tukey’s HSD test.

**Table 2:** Phenolic compound profile of sage roots treated with LED light.

Treatment	Individual Phenolic Compounds (µg/g)			
	Chlorogenic Acid	Caffeic Acid	Ellagic Acid	Rosmarinic Acid
Roots maintained in darkness	1.535 ± 0.021 ^a^	0.57 ± 0.003 ^a^	ND	0.841 ± 0.006 ^c^
Roots illuminated with white light	0.915 ± 0.019 ^b^	0.86 ± 0.011 ^a^	ND	1.581 ± 0.023 ^b^
Roots illuminated with blue light	ND	0.138 ± 0.002 ^c^	ND	ND
Roots illuminated with red light	ND	0.29 ± 0.014 ^b^	ND	2.873 ± 0.021 ^a^
Roots illuminated with green light	ND	0.163 ± 0.011 ^c^	0.863 ± 0.007	3.099 ± 0.002 ^a^

Values are presented as means ± standard deviation from 3 independent biological replicates. ND, not detected. Different superscript letters indicate statistically significant differences among treatments according to Tukey’s HSD test.

**Table 3:** Effect of treatment with chemical elicitors (salicylic acid and AgNO_3_) on the level of total polyphenols in hairy roots of *S. officinalis*.

Treatment	Total Polyphenol Content (mg GAE/g Roots or mg GAE/mL Supernatant)		
	Incubation Period		
	0 Days (Untreated Control)	4 Days	7 Days
Salicylic acid	0.701 ± 0.018 ^c^	0.513 ± 0.012 ^d^	0.313 ± 0.002 ^e^
AgNO_3_	0.701 ± 0.017 ^c^	0.938 ± 0.019 ^b^	0.976 ± 0.013 ^a^

Values are presented as means ± standard deviation from 3 independent biological replicates. Different superscript letters indicate statistically significant differences among treatments according to Tukey’s HSD test.

**Table 4:** Phenolic compound profile of sage roots treated with chemical elicitors.

Treatment	Incubation Period (Days)	Individual Phenolic Compounds (µg/g)							
		Gallic Acid	Chlorogenic Acid	Caffeic Acid	Syringic Acid	*p*-Coumaric Acid	Rutin	Rosmarinic Acid	Quercetin
Roots treated with salicylic acid	4	0.300 ± 0.012 ^c^	0.257 ± 0.002 ^d^	1.608 ± 0.011 ^c^	0.133 ± 0.002 ^c^	ND	0.186 ± 0.002 ^d^	0.327 ± 0.003 ^c^	ND
	7	0.130 ± 0.005 ^d^	0.46 ± 0.015 ^c^	ND	ND	ND	0.388 ± 0.007 ^c^	0.151 ± 0.002 ^d^	ND
Roots treated with AgNO_3_	4	0.560 ± 0.013 ^b^	2.787 ± 0.019 ^a^	11.65 ± 0.018 ^b^	3.464 ± 0.015 ^a^	0.178 ± 0.01	1.518 ± 0.008 ^a^	6.248 ± 0.009 ^b^	0.137 ± 0.002
	7	0.800 ± 0.019 ^a^	2.511 ± 0.012 ^b^	17.08 ± 0.015 ^a^	0.959 ± 0.002 ^b^	ND	0.977 ± 0.002 ^b^	7.934 ± 0.013 ^a^	ND

Values are presented as means ± standard deviation from 3 independent biological replicates. ND, not detected. Different superscript letters indicate statistically significant differences among treatments according to Tukey’s HSD test.

**Table 5:** Phenolic compound content of roots treated with biotic elicitors.

Sample		Total Polyphenol Content (mg GAE/g Roots or mg GAE/mL Supernatant)		
		Day 0	Day 7	Day 14
**Biologically elicited root extracts**				
*B. velezensis* BPVs2	Cell lysate	0.701 ± 0.012 ^c^	0.363 ± 0.015 ^e^	0.413 ± 0.013 ^d^
	0.75 mL cell-free supernatant	0.701 ± 0.012 ^c^	0.563 ± 0.012 ^d^	0.338 ± 0.009 ^e^
	1.5 mL cell-free supernatant	0.701 ± 0.012 ^c^	0.988 ± 0.007 ^b^	0.313 ± 0.006 ^e^
*T. asperellum* TdCP	Biomass lysate	0.701 ± 0.012 ^c^	1.363 ± 0.019 ^a^	0.488 ± 0.011 ^d^
**Supernatant from biologically elicited root cultures**				
*B. velezensis* BPVs2	Cell lysate	0.012 ± 0.001 ^c^	0.016 ± 0.002 ^b^	0.016 ± 0.002 ^b^
	0.75 mL cell-free supernatant	0.012 ± 0.001 ^c^	0.022 ± 0.001 ^a^	0.027 ± 0.003 ^a^
	1.5 mL cell-free supernatant	0.012 ± 0.001 ^c^	0.025 ± 0.002 ^a^	0.026 ± 0.001 ^a^
*T. asperellum* TdCP	Biomass lysate	0.012 ± 0.001 ^c^	0.016 ± 0.001 ^b^	0.017 ± 0.001 ^b^

Values are presented as means ± standard deviation from 3 independent biological replicates. Different superscript letters indicate statistically significant differences among treatments according to Tukey’s HSD test.

**Table 6:** Phenolic compound profile of sage roots treated with biotic elicitors.

Treatment	Incubation Period (Days)	Individual Phenolic Compounds (µg/g)						
		Gallic Acid	Chlorogenic Acid	Caffeic Acid	Syringic Acid	*p*-Coumaric Acid	Rutin	Rosmarinic Acid
**Roots elicited with *B. velezensis***								
Cell lysate	7	1.397 ± 0.022 ^a^	0.582 ± 0.011 ^b^	2.916 ± 0.025 ^b^	0.313 ± 0.005 ^c^	0.054 ± 0.002 ^a^	0.336 ± 0.001 ^c^	0.594 ± 0.003 ^c^
	14	0.4 ± 0.011 ^d^	0.784 ± 0.019 ^a^	2.586 ± 0.019 ^c^	0.488 ± 0.006 ^b^	<LoQ	0.478 ± 0.006 ^b^	0.323 ± 0.001 ^d^
0.75 mL cell-free supernatant	7	<LoQ	0.370 ± 0.023 ^c^	3.006 ± 0.020 ^b^	0.42 ± 0.005 ^b^	ND	0.374 ± 0.009 ^c^	0.920 ± 0.009 ^b^
	14	0.132 ± 0.014 ^e^	0.128 ± 0.015 ^e^	0.984 ± 0.013 ^d^	ND	ND	ND	0.779 ± 0.018 ^b^
1.5 mL cell-free supernatant	7	0.378 ± 0.011 ^d^	0.522 ± 0.018 ^b^	9.798 ± 0.02 ^a^	0.896 ± 0.009 ^a^	ND	1.960 ± 0.013 ^a^	25.635 ± 0.029 ^a^
	14	0.246 ± 0.018 ^e^	0.304 ± 0.001 ^d^	0.745 ± 0.007 ^e^	ND	ND	ND	0.468 ± 0.016 ^c^
**Roots elicited with *T. asperellum***								
TdCP Biomass lysate	7	0.842 ± 0.001 ^c^	2.961 ± 0.018 ^a^	2.961 ± 0.018 ^b^	0.487 ± 0.002 ^b^	0.097 ± 0.002 ^a^	ND	1.265 ± 0.015 ^b^
	14	0.554 ± 0.004 ^d^	1.574 ± 0.002 ^b^	1.575 ± 0.021 ^c^	ND	0.050 ± 0.001 ^a^	ND	0.327 ± 0.008 ^d^

Values are presented as means ± standard deviation from 3 independent biological replicates. ND, not detected; LoQ, limit of quantification. Different superscript letters indicate statistically significant differences among treatments according to Tukey’s HSD test.

**Table 7:** Inhibitory effect of some extracts of *S. officinalis* hairy roots on *B. spizizenii* ATCC 6633.

Elicitor	Antibacterial Effect Against *B. spizizenii* ATCC 6633
**Sage-root extracts**	
Extract from untransformed roots	±
Hairy-root extract, control	±
AgNO_3_	++
*B. velezensis* BPVs2 cell lysate	+
0.75 mL bacterial cell-free supernatant	+
1.5 mL bacterial cell-free supernatant	++
*T. asperellum* TdCP biomass lysate	+
**Supernatants from elicited sage-root cultures**	
AgNO_3_	−
*B. velezensis* BPVs2 cell lysate	−
0.75 mL bacterial cell-free supernatant	−
1.5 mL bacterial cell-free supernatant	−
*T. asperellum* TdCP biomass lysate	−

Legend: −, no antibacterial activity; ±, low inhibitory activity, bacteriostatic, manifested as a reduction in the density of the target bacterial cells; +, low antibacterial activity; ++, moderate antibacterial activity.

**Table 8:** Testing the antifungal activity of sage root extracts subjected or not subjected to elicitation.

Samples	Elicitor Used	Inhibitory Effect of Root Extracts Against Filamentous Fungi		
		*Sclerotinia sclerotiorum*	*Fusarium* sp.	*Aspergillus* sp.
Untransformed-root extract, control	−	−	−	−
Hairy-root extract, control	−	±	−	−
Hairy-root extract	AgNO_3_	++	±	−
Hairy-root extract	*B. velezensis* BPVs2 cell lysate	+	±	−
Hairy-root extract	0.75 mL bacterial cell-free supernatant	+	±	−
Hairy-root extract	1.5 mL bacterial cell-free supernatant	++	+	±
Hairy-root extract	*T. asperellum* TdCP biomass lysate	+	−	−

Legend: −, no antifungal activity; ±, low inhibitory activity; +, low antifungal activity; ++, moderate antifungal activity.

## Data Availability

The original contributions presented in this study are included in the article. Further inquiries can be directed to the corresponding author.

## Abbreviations

The following abbreviations are used in this manuscript:

ANOVA	Analysis of variance
ATCC	American Type Culture Collection
CFBS	Cell-free bacterial supernatant
GAE	Gallic acid equivalents
HPLC	High-performance liquid chromatography
LB	Luria–Bertani medium
LC-HRMS	Liquid chromatography high-resolution mass spectrometry
LED	Light-emitting diode
LoQ	Limit of quantification
MICs	Minimum inhibitory concentrations
MRSA	Methicillin-resistant Staphylococcus aureus
MS	Murashige and Skoog medium
ND	Not detected
NCAIM	Microorganism collection code, not expanded in the manuscript
PAL	Phenylalanine ammonia-lyase
PCR	Polymerase chain reaction
PDA	Potato Dextrose Agar
ROS	Reactive oxygen species
SA	Salicylic acid
